# Fingerprint of Circulating Immunocytes as Biomarkers for the Prognosis of Brain Inflammation and Neuronal Injury after Cardiac Arrest

**DOI:** 10.1021/acschemneuro.3c00397

**Published:** 2023-11-15

**Authors:** Huanyu Dou, Nicole R. Brandon, Kerryann E. Koper, Yan Xu

**Affiliations:** † Department of Molecular and Translational Medicine, Paul L. Foster School of Medicine, and Graduate School of Biomedical Sciences, Texas Tech University Health Science Center, El Paso, Texas 79905, United States; ‡ Departments of Anesthesiology and Perioperative Medicine, Pharmacology and Chemical Biology, and Structural Biology, University of Pittsburgh School of Medicine, Pittsburgh, Pennsylvania 15213, United States; § Department of Physics and Astronomy, The Dietrich School of Arts and Sciences, University of Pittsburgh, Pittsburgh, Pennsylvania 15213, United States; ∥ Departments of Anesthesiology and Perioperative Medicine, University of Pittsburgh School of Medicine, Pittsburgh, Pennsylvania 15213, United States

**Keywords:** cardiac arrest and resuscitation, blood biomarkers, global ischemia, *T*_reg_, polymorphonuclear myeloid-derived suppressor cells (PMN-MDSCs), peripheral–central immune coupling

## Abstract

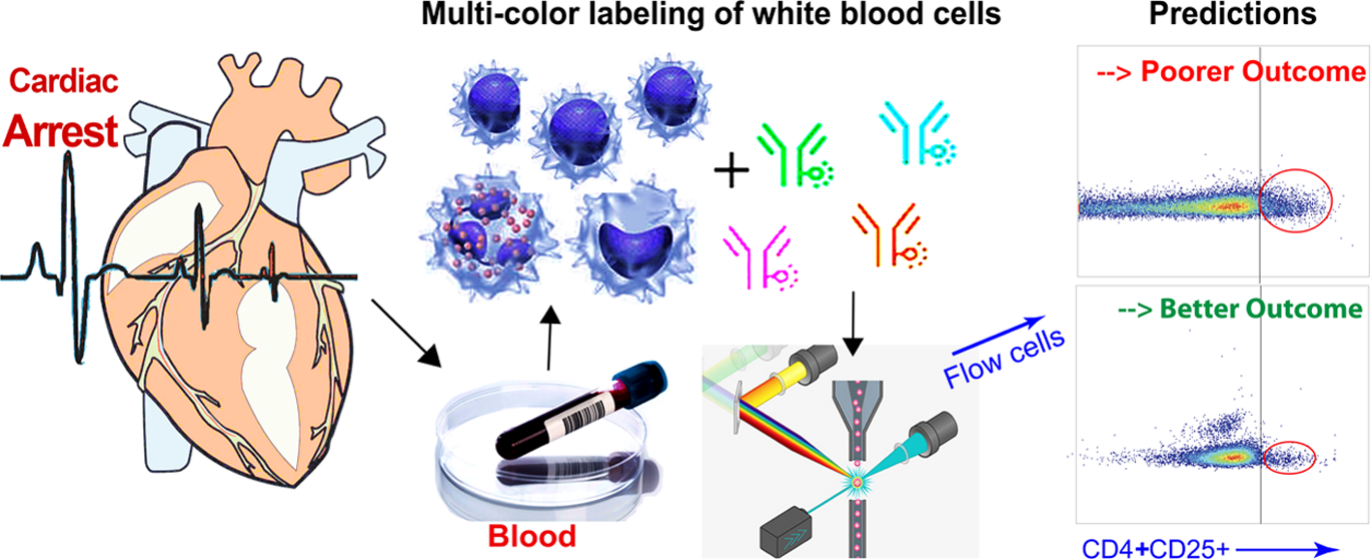

Cardiac arrest is one of the most dangerous health problems in the world. Outcome prognosis is largely based on cerebral performance categories determined by neurological evaluations. Few systemic tests are currently available to predict survival to hospital discharge. Here, we present the results from the preclinical studies of cardiac arrest and resuscitation (CAR) in mice to identify signatures of circulating immune cells as blood-derived biomarkers to predict outcomes after CAR. Two flow cytometry panels for circulating blood lymphocytes and myeloid-derived cells, respectively, were designed to correlate with neuroinflammation and neuronal and dendritic losses in the selectively vulnerable regions of bilateral hippocampi. We found that CD4^+^CD25^+^ regulatory T cells, CD11b^+^CD11c^–^ and CD11b^+^Ly6C^+^Ly6G^+^ myeloid-derived cells, and cells positive for the costimulatory molecules CD80 and CD86 in the blood were correlated with activation of microglia and astrocytosis, and CD4^+^CD25^+^ T cells are additionally correlated with neuronal and dendritic losses. A fingerprint pattern of blood T cells and monocytes is devised as a diagnostic tool to predict CAR outcomes. Blood tests aimed at identifying these immunocyte patterns in cardiac arrest patients will guide future clinical trials to establish better prognostication tools to avoid unnecessary early withdrawal from life-sustaining treatment.

## Introduction

Worldwide, over 4.4 million people suffer from out-of-hospital cardiac arrest each year.^1^ Despite the steadily improved cardiopulmonary resuscitation guidelines, the rate of survival to hospital discharge remains unacceptably low, at 2–14%.^2−5^ Most deaths occur as the result of withdrawing life-sustaining treatment following pessimistic prognostication based largely on the cerebral performance categories (CPC) of predicted neurological outcomes.^6,7^ Few systemic evaluation tools are currently available for accurately predicting outcomes after cardiac arrest and resuscitation (CAR).

CAR triggers a systemic immune reaction, leading to a cascade of deteriorating changes in the brain.^8^ Innate and adaptive immune responses to circulatory arrest have been well documented in both clinical and experimental settings,^9−12^ and systemic immune changes are often linked to neurological complications after CAR.^13,14^ In contrast to acute focal ischemia, in which a robust immune response to brain injury can occur even when immune cells are not found in the brain tissues,^15,16^ the injury pattern after global ischemia is distinctly different. Hence, the immune responses under focal ischemia and global ischemia should not be assumed to be the same. Little is currently known about the roles of circulating blood immunocytes in neuronal injuries after CAR. Upon resuscitation, the brain exhibits protracted hypoperfusion beyond the immediate reperfusion period,^17^ with accompanied irregularity in cerebral metabolisms.^18−20^ The cerebral metabolic stress is believed to impact the immune and inflammatory responses, leading to different degrees of neuronal injuries and outcomes.^21^ Research into the predictive roles of systemic immune biomarkers in the circulating blood will provide new diagnostic tools to supplement the current use of CPC scales for better prognostication of the cardiac arrest outcome in a clinical setting.

In this study, we used a clinically relevant CAR model in mice to investigate the relationship between circulating blood immunocytes and the pathological changes of neuronal damage and neuroinflammation. Classification of possible correlations is essential for identifying cellular markers in the circulating blood that link to the prognosis of neuropathological outcomes. By combining flow cytometry and histological assays, we show that the levels of neuronal injuries are correlated with the circulating CD4^+^CD25^+^ regulatory T cells, CD11b^+^CD11c^–^ monocytes, CD11b^+^Ly6C^+^Ly6G^+^ polymorphonuclear myeloid-derived suppressor cells (PMN-MDSCs), and cells expressing the costimulatory molecules CD80 and CD86 in the blood. These subpopulations of blood cells were up- or downregulated and associated with the severity of neuronal damage and neuroinflammation after CAR.

## Results

### Brain Immune Responses to Cardiac Arrest and Resuscitation

**Figure 1:**
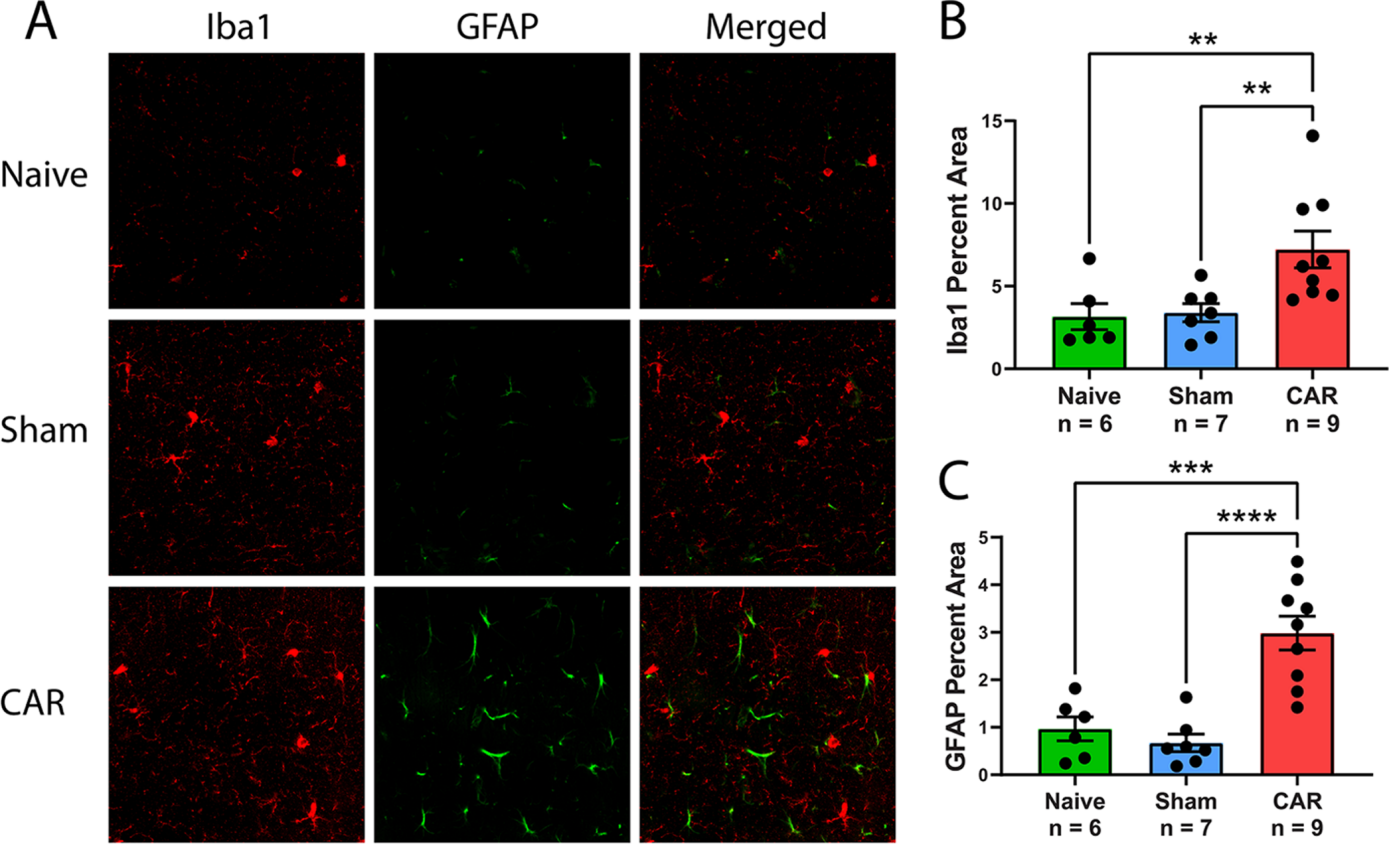
Neuroinflammation after cardiac arrest and resuscitation (CAR). (A) Representative images of Iba1 (red) and GFAP (green) staining in the hippocampal CA1 region of mice from each group. (B) Percent area of Iba1 positive staining in the hippocampal CA1 region is plotted by the group. Values from individual mice are displayed by black circles. Bars show mean ± standard error of the mean (SEM) (*n* = 6–9). One-way analysis of variance (ANOVA) shows significant differences among all groups (*p* = 0.007). Fisher’s post hoc least significant difference (LSD) comparisons show significant differences between naïve and CAR groups (*p* = 0.006, indicated by **) and between sham and CAR groups (*p* = 0.007). (C) Percent area of GFAP-positive staining for the same CA1 regions of interest as for Iba1. One-way ANOVA gives *p* < 0.0001. Fisher’s post hoc LSD comparisons show *p* = 0.0002 (indicated by ***) between naïve and CAR groups and *p* < 0.0001 (indicated by ****) between sham and CAR groups.

Activation of Iba1^+^ microglia and GFAP^+^ astrocytes is a signature of inflammatory neuropathological changes in the brain. Double fluorescence labeling of Iba1 and GFAP in brain sections was used to determine the activation of microglia and reactive astrocytosis. Imaging of brain sections showed increases in Iba1^+^ cells with large, ramified microglia in CAR mice compared to naïve and sham-operated animals (Figure 1A, red). Morphometric analysis was used to calculate the Iba1^+^ microglial area as a percentage of the total area, measured by the positively stained pixels and total pixels in the region of interest (ROI) as defined in the Methods and Materials section. The Iba1^+^ microglia in the CA1 area of the hippocampus, with ROI extending from the corpus callosum to the dentate gyrus, significantly increased in mice with CAR compared to the naïve and sham-operated mice (*p* = 0.0064 and 0.0071, respectively, Figure 1B). There were no significant differences between naïve and sham-operated mice. We costained GFAP to determine astrocyte activation. Manifestation of GFAP^+^ astrocytes was clearly visible in the brains after CAR. GFAP^+^ astrocytes had typically enlarged cell bodies with a reactive morphology. The activation of GFAP^+^ astrocytes with these properties was observed in the CA1 region of the hippocampus (Figure 1A, green). The percentages of the GFAP-positive area were calculated to quantify the activation of astrocytes (Figure 1C). There was a significant increase in GFAP^+^ staining in CAR mice compared to the naïve (*p* = 0.0002) and sham-operated controls (*p* < 0.0001, Figure 1C). A slight decrease in GFAP^+^ staining was seen in the sham-operated mice, but the difference was not statistically significant between the sham and naïve animals.

### Neuronal Injury after Cardiac Arrest and Resuscitation

**Figure 2:**
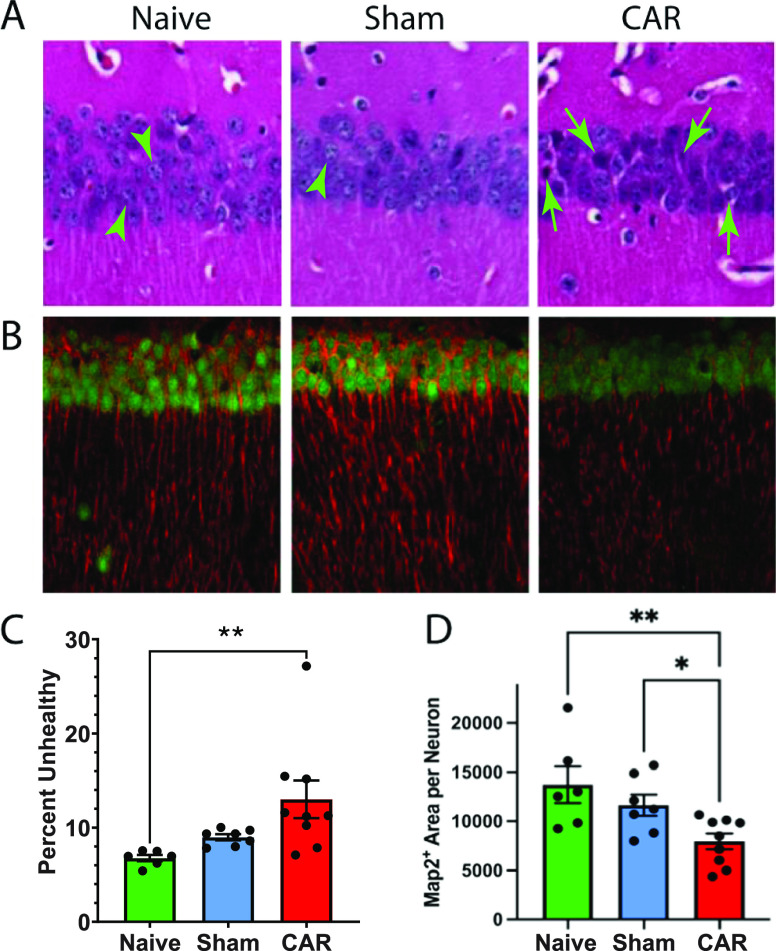
Neuron and dendrite losses after cardiac arrest and resuscitation (CAR). (A) H&E staining of the pyramidal neurons in the hippocampal CA1 region from mice in the three experimental groups. Neuronal counting was performed in a blind fashion in four predetermined regions of interest along the pyramidal neuron band of the CA1 region. Arrowheads point to examples of healthy neurons, whereas arrows with stems indicate examples of various types of damaged neurons. (B) Micrographs of Map2 (red) and Neun (green) costaining in the CA1 region showing damage to the dendritic processes in the CAR group. (C, D) Bar graphs summarizing neuron and dendrite losses in the hippocampal CA1 region. Each black circle represents one mouse. Error bars show SEM (*n* = 6–9), with * and ** signifying *p* < 0.05 and <0.01, respectively, by one-way ANOVA followed by Fisher’s LSD post hoc comparisons.

CAR-induced neuronal injury was evaluated in the hippocampal CA1 region, where the neurons are selectively vulnerable to global ischemia in mouse and rat CAR models.^8,22−25^ Unlike neuronal nuclear marker (NeuN^+^) counting, which identifies neuronal nuclei of both injured and uninjured neurons, histological assessment of the hippocampal CA1 neurons by hematoxylin and eosin (H&E) staining can reliably distinguish healthy and unhealthy neurons based on hyperbasophilia, perinuclear vacuolization, and shrinkage and dysmorphic shape of the nuclei. The healthy (Figure 2A, green arrowheads) and unhealthy (Figure 2A, green arrows) neurons were counted in four predetermined ROIs in the CA1 region of the hippocampi in both hemispheres for each animal. Neuronal injury was quantified by calculating the percentage of unhealthy neurons in the total number of neurons in all of the ROIs (Figure 2C). The percentage of unhealthy neurons significantly increased by day 5 after CAR (*p* = 0.0089) compared to the naïve and sham-operated groups. One of the clinical characteristics in CPC evaluation of cardiac arrest patients is the large variations from seemingly similar cardiac arrest episodes. This large variation is mirrored in the experimental CAR models by the widely varying number of unhealthy neurons in the CA1 region. Indeed, ∼20% of the CAR mice showed similar histology scores to the naïve and sham-operated mice. Significant neuronal injuries appeared in ∼70% of CAR mice, whereas ∼10% of CAR mice showed substantially increased numbers of unhealthy neurons in the CA1 region.

To quantify neuronal injuries by the loss of neuronal processes, we used dual fluorescence labeling of microtubule-associated protein 2 (Map2, Figure 2B, red) and NeuN (Figure 2B, green) to measure the density of s from neuronal cell bodies. Quantitative imaging analysis by threshold segmentation was used to evaluate the loss of Map2^+^ dendrites normalized by the number of NeuN^+^ neuronal nuclei in the CA1 region (Figure S3A,B). After normalizing by a comparable number of neurons among three groups based on NeuN staining in the ROI, Figure 2B shows that the Map2^+^ density and dendrite lengths significantly decrease in the CAR mice as compared with the naïve and sham-operated animals (*p* = 0.003 and 0.035, respectively). In contrast, no significant differences in Map2^+^ immunoreactivity are found between naïve and sham-operated mice.

Similar to H&E neuronal counting, ∼22% of CAR mice had a comparable level of dendrite density to the naïve and sham-operated mice (Map2^+^/Neurons >10,000 units), and ∼23, ∼45, and ∼10% CAR mice showed mild [within one standard deviation (SD) of the naïve and sham average], moderate (between 1 and 2 SD), and severe (outside 2 SD) loss of dendritic processes, respectively. These categorical differences in neuronal injury scores are used to correlate with changes in the circulating immunocytes, as detailed below.

### Identification of Circulating Blood T Cell Differentiation

The blood collected from naïve, sham-operated, and CAR mice was used to isolate white blood cells. Flow cytometry analysis with the gating strategy as shown in Figure S1 was used to identify and quantify CD45^+^, CD3^+^, CD8^+^, CD4^+^, CD25^+^, and CD28^+^ T cells in the circulating blood.

**Figure 3:**
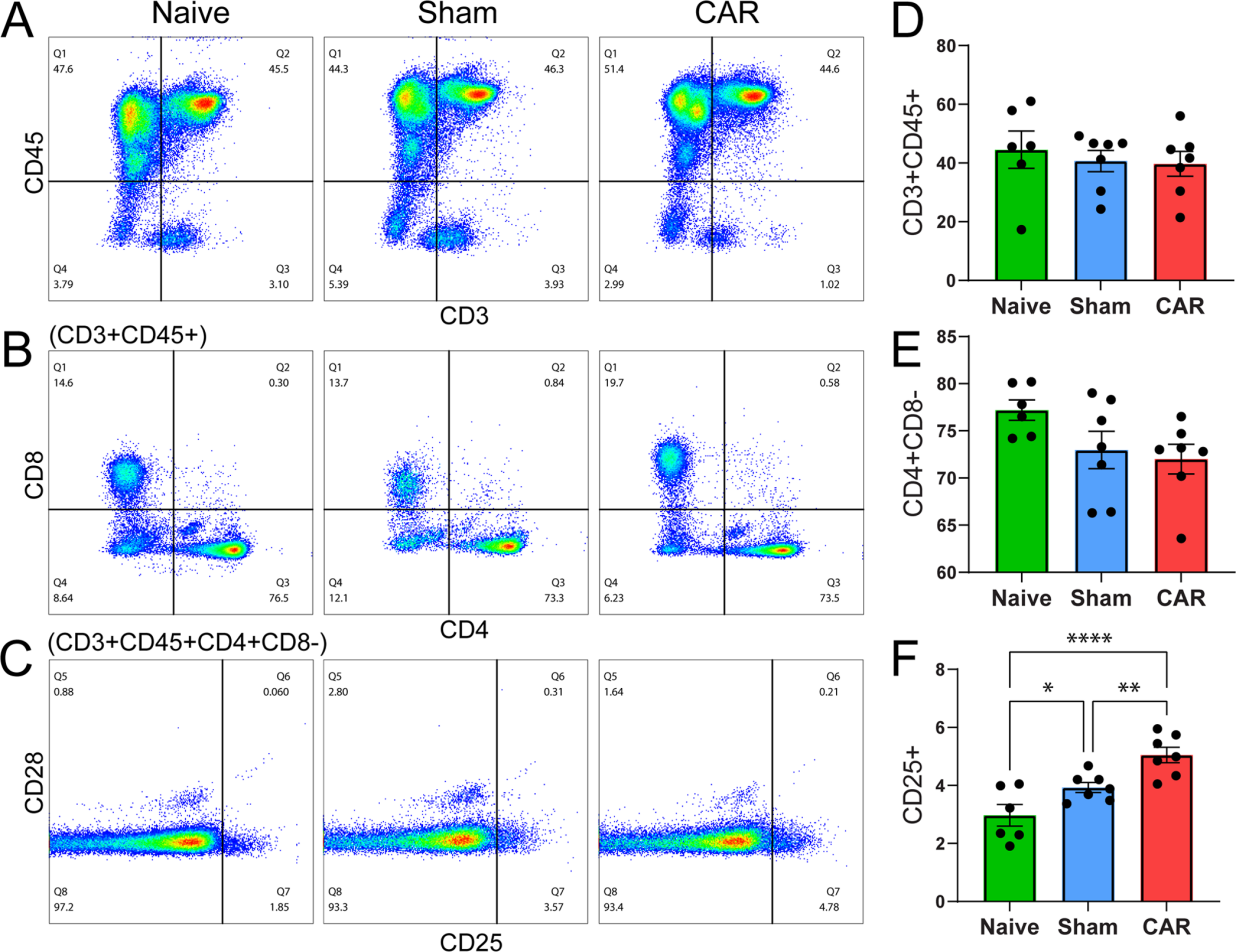
Analysis of circulating blood T cells by flow cytometry. Blood flow cytometry data, gated as shown in Figure S1, are plotted for (A) CD3 versus CD45, (B) CD4 versus CD8 from the CD3^+^CD45^+^ subpopulation, and (C) CD25 versus CD28 from the CD3^+^CD45^+^CD4^+^CD8^–^ subpopulation. (D–F) Corresponding bar graphs representing the indicated gate for each mouse are displayed to the right of respective flow plots. Each black circle represents an individual mouse, and error bars show SEM (*n* = 6–7). *, **, and **** indicate *p* < 0.05, < 0.01, and < 0.0001, respectively, by Fisher’s LSD multiple comparisons in a one-way ANOVA design.

Antibody Panel 1 (Table S2) was used to analyze white blood cells and to distinguish T cell subpopulations. Representative plotting analyses of circulating lymphocytes in the blood with antibodies specific for CD45^+^, CD3^+^, CD4^+^, CD8^+^, and CD25^+^ are presented in Figure 3A–C. Averaged subpopulations among animals in each group are summarized in Figure 3D–F, with each animal represented by a black dot. Two Panel 1 samples in the CAR group had technical errors in the flow cytometry runs and were excluded from Panel 1 analyses. The CD3^+^CD45^+^ T cells are not significantly different among naïve, sham-operated, and CAR groups (Figure 3A,D), and the CD4- and CD8-gated CD3^+^CD45^+^ T cells were present in lower frequency for the CD4^+^CD8^–^ subpopulation in the CAR mice (Figure 3B,E). No significant difference was observed in the subsets of CD4^+^ T cells between the naïve and sham-operated mice. However, when we analyzed CD25 markers on CD4^+^ subpopulations, CD4^+^CD25^+^ T cells were significantly elevated (Figure 3E,F) in CAR mice compared to naïve mice (*p* < 0.0001) and sham-operated controls (*p* = 0.0086). An increase in the frequency of CD4^+^CD25^+^ T cells indicated an upregulation in the suppressive *T*_reg_ cells in the circulating blood 5 days after CAR in mice. CD25 expression on CD4^+^ T cells was also elevated in sham-operated mice compared to the naïve control (*p* = 0.026) (Figure 3F).

### Distinct Subpopulations of Innate Immune Cells in Circulating Blood

**Figure 4:**
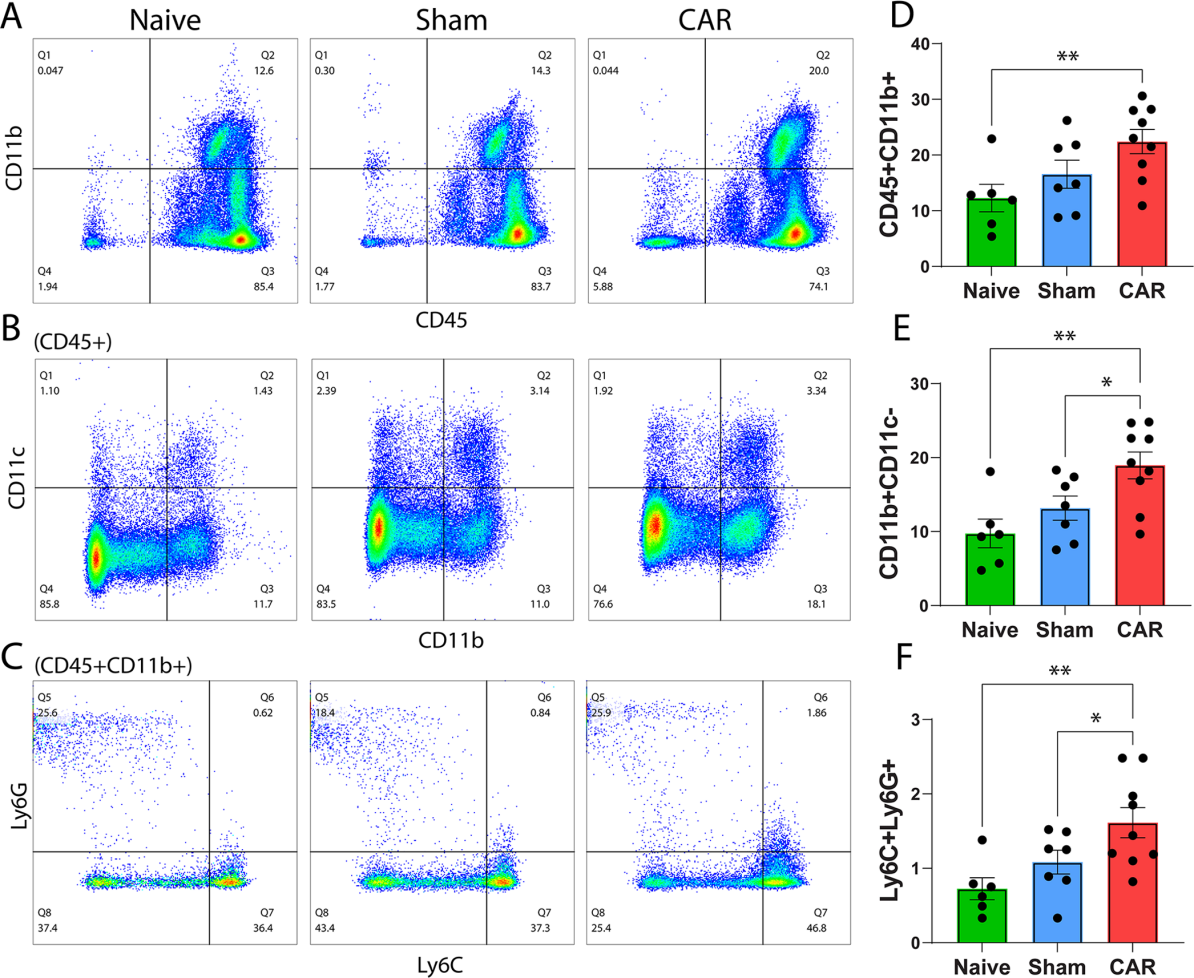
Analysis of circulating blood monocytes by flow cytometry. Blood flow cytometry data, gated as in Figure S2, are plotted for (A) CD45 versus CD11b, (B) CD11b versus CD11c in the CD45^+^ subpopulation, and (C) Ly6C versus Ly6G in the CD45^+^CD11b^+^ subpopulation. (D–F) Corresponding bar graphs representing the indicated quadrant gate for each mouse are displayed to the right of their corresponding flow cytometry plots. Each black circle represents an individual mouse, and error bars show SEM (*n* = 6–9). * and ** indicate *p* < 0.05 and < 0.01, respectively, by Fisher’s LSD multiple comparisons in a one-way ANOVA design.

Antibody Panel 2 in Table S2 was used to identify monocytes and granulocytes in the circulating blood with the flow cytometry gating strategy as in Figure S2. To determine whether CAR regulates circulating blood monocyte and granulocyte polarization, we characterized innate immune cells with antibodies against CD45, CD11b, CD11c, Ly6C, and Ly6G. The gating strategy successfully isolated the subsets of monocytes and granulocytes in circulating blood and revealed their relative proportions. The CD45^+^CD11b^+^ granulocytes and monocytes were 12 ± 6% in naïve animals, 17 ± 7% in the sham-operated group, and increased to 22 ± 7% in the CAR mice. This increase in the CD45^+^CD11b^+^ subset (Figure 4A,D) was statistically significant compared to the naïve (*p* = 0.0077) but not the sham-operated controls (*p* = 0.0882). To further discern CD11b^+^ cells, three subsets were analyzed based on the expression of CD11c, Ly6C, and Ly6G. We first gated blood CD11c^+^ dendritic cells (DCs) in the CD45^+^CD11b^+^ populations, and the CD11b^+^CD11c^–^, CD11b^+^CD11c^+^, and CD11b^–^CD11c^+^ subpopulations were identified (Figure 4B). While the CD45^+^CD11c^+^ population was not significantly different among the three groups, a significant upregulation was found for the CD11b^+^CD11c^–^ subpopulation after CAR (Figure 4B), averaging 19.0 ± 1.8% in the CAR group, compared to 9.7 ± 1.9 and 13.2 ± 1.6% in the naïve and sham controls, respectively (Figure 4E). We further gated CD45^+^CD11b^+^ cells by Ly6C and Ly6G. Three subpopulations were identified with CD11b^+^Ly6C^+^Ly6G^–^ monocytes, CD11b^+^Ly6C^–^Ly6G^+^ granulocytes, and CD11b^+^Ly6C^+^Ly6G^+^ PMN-MDSCs (Figure 4C). CD11b^+^Ly6C^+^Ly6G^+^ immune suppressive cells represented a small population, but these cells increased significantly (Figure 4C,F) in the CAR mice (1.6 ± 0.2%) compared to the naïve (0.7 ± 0.1%, *p* = 0.0031) and sham-operated controls (1.1 ± 0.2%, *p* = 0.047).

### Correlation between Neuroinflammation and Immunomarkers in the Circulating Blood Cells

**Figure 5:**
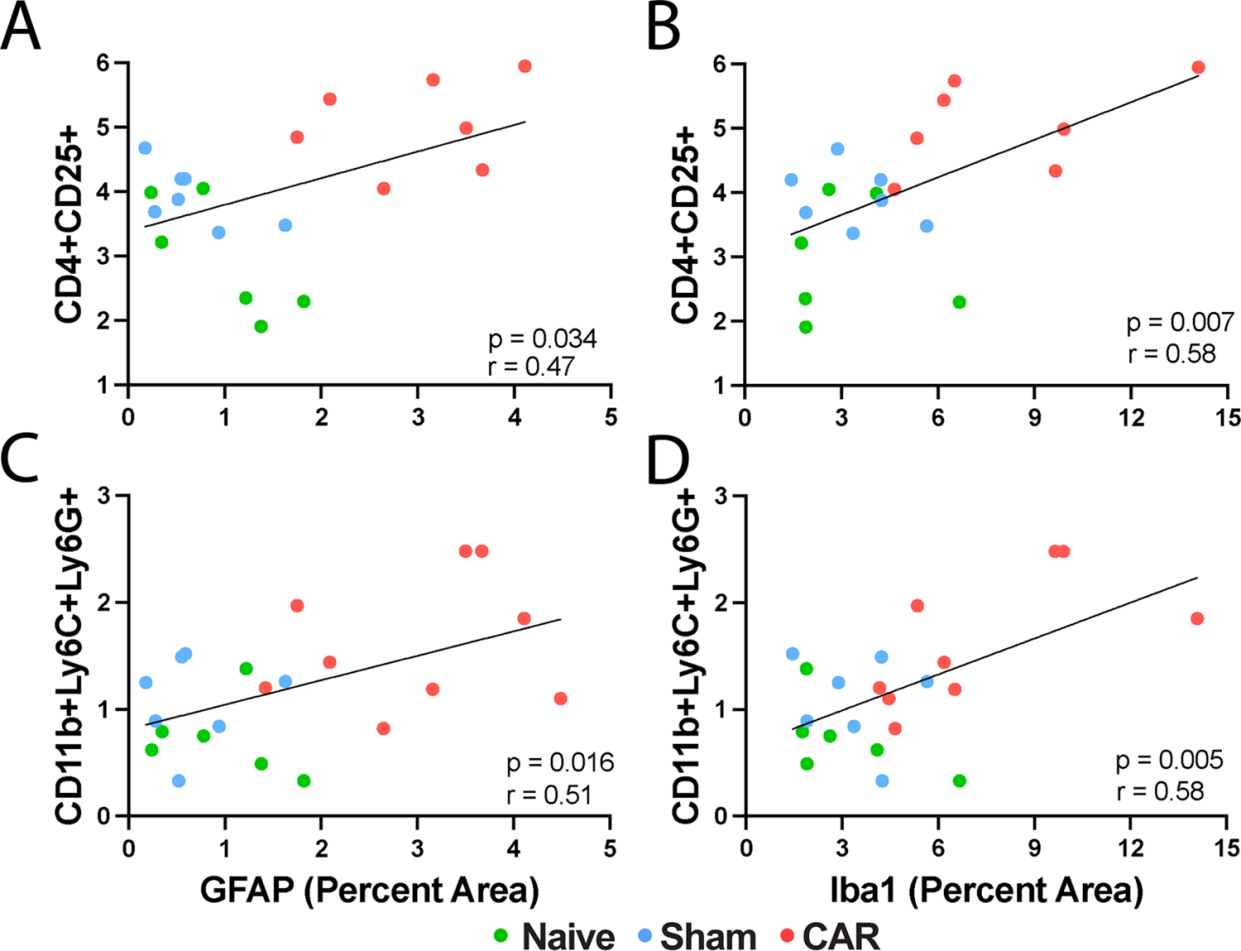
Correlation between neuroinflammation and circulating blood cell markers. Subpopulations of CD4^+^CD25^+^ T cells (A, B) and CD11b^+^Ly6C^+^Ly6G^+^ PMN-MDSCs (C, D) in the circulating blood are correlated with the activation of GFAP^+^ astrocytes (A, C) and Iba1^+^ microglia (B, D) in the hippocampal CA1 region. Each colored circle represents a mouse from the naïve (green, *n* = 6), sham (cyan, *n* = 7), or CAR (red, *n* = 7 or 9 for Antibody Panel 1 or 2, respectively) group. The solid lines depict the Pearson correlation with the *p*-value and correlation coefficient r as indicated. The coefficients of determination, *R*^2^, are (A) 0.22, (B) 0.34, (C) 0.26, and (D) 0.34.

To further determine whether the changes in circulating blood immunocytes under normal and pathological conditions due to CAR corresponded with neuroinflammation as measured by the activation of microglia and astrocytes (see Figure 1), we performed quantitative analysis in the hippocampal CA1 regions to determine whether the activated Iba1^+^ microglia and GFAP^+^ astrocytes were correlated with the changes in circulating blood CD4^+^CD25^+^ T cells and CD11b^+^Ly6C^+^Ly6G^+^ PMN-MDSCs or the neuroinflammation was independent of the changes in circulating immunocytes in the blood. A correlative analysis between parameters for the central and peripheral immunomarkers is presented in Figure 5. We found that the increases in GFAP^+^ astrocytes in CAR mice compared to the naïve and sham controls were positively correlated with both CD4^+^CD25^+^ T cells (Figure 5A, *p* = 0.0344) and CD11b^+^Ly6C^+^Ly6G^+^ MDSCs (Figure 5C, *p* = 0.0156). Moreover, we observed that upregulated Iba1^+^ microglia immunoreactivity in the hippocampal CA1 area is also strongly and positively correlated with CD4^+^CD25^+^ T cells (Figure 5B, *p* = 0.0069) and CD11b^+^Ly6C^+^Ly6G^+^ immune suppressive cells (Figure 5D, *p* = 0.0050) in the circulating blood. The elevation of circulating blood subpopulations of CD4^+^CD25^+^ T cells and CD11b^+^Ly6C^+^Ly6G^+^ cells thus can serve as circulating biomarkers to indicate the levels of neuroinflammation measured by the increases in Iba1^+^ microglial and GFAP^+^ reactive astrocytosis in the brain.

### Correlation between Neural Damage and Circulating Blood Cells

**Figure 6:**
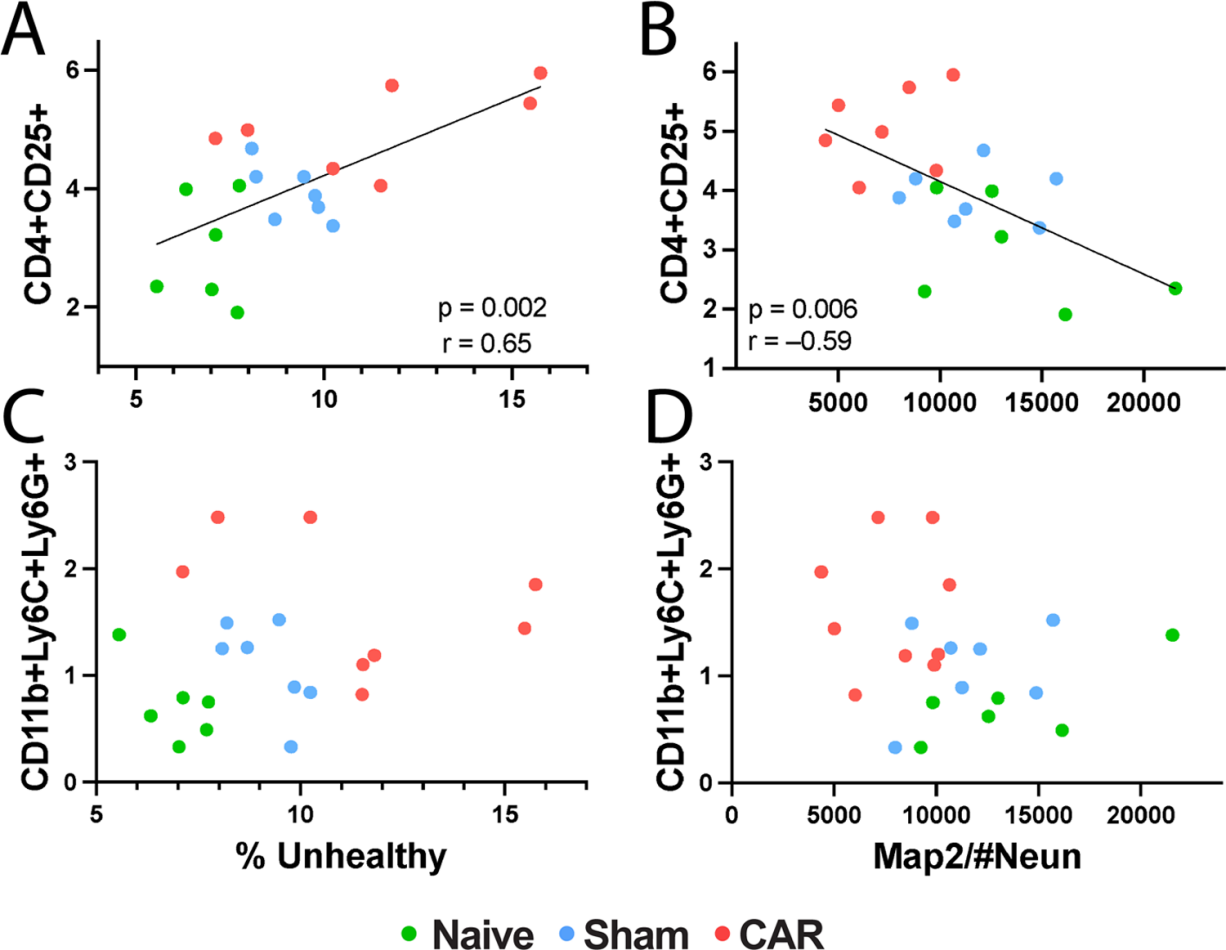
Correlation between neuronal injuries and circulating blood cell markers. The subpopulation of CD4^+^CD25^+^ T cells in the circulating blood is strongly correlated with the percent unhealthy neurons (A) and dendritic loss measured by Map2 density/neuron (B) in the hippocampal CA1 region. Each colored circle represents a mouse from the naïve (green, *n* = 6), sham (cyan, *n* = 7), or CAR (red, *n* = 7 or 9 for Antibody Panel 1 or 2, respectively) group. The solid lines depict the Pearson correlation with the *p*-value and correlation coefficient *r* as indicated. In contrast, the subpopulation of CD11b^+^Ly6C^+^Ly6G^+^ PMN-MDSCs is not significantly correlated with neuronal loss (C, *p* = 0.56) and dendritic loss (D, *p* = 0.09, *r* = −0.37).

To determine whether the circulating blood cells can serve as biomarkers to predict neuronal damage, we correlated the upregulated subpopulations of CD4^+^CD25^+^ T cells (Figure 3C,F) and CD11b^+^Ly6C^+^Ly6G^+^ PMN-MDSCs (Figure 4) with the counts of H&E-stained unhealthy neurons and the loss of Map2^+^ neurites (Figure 2). These correlations are plotted in Figure 6 as functions of the number of unhealthy neurons and Map2^+^ staining. Neuronal loss in the hippocampal CA1 region was positively correlated with blood CD4^+^CD25^+^ T cells (Figure 6A, *p* = 0.0019) but not with CD11b^+^Ly6C^+^Ly6G^+^ PMN-MDSCs (Figure 6C, *p* = 0.6125). Neuron-normalized Map2^+^ neurites in the hippocampus showed a strong negative correlation with the CD4^+^CD25^+^ regulatory T cells in the circulating blood (Figure 6B) but not with the CD11b^+^Ly6C^+^Ly6G^+^ PMN-MDSCs (Figure 6D).

Correlations shown in Figures 5 and 6 suggest that the severity of neural injury in the brain can be linked to markers in the circulating blood, particularly the CD4^+^CD25^+^ T cells and CD11b^+^Ly6C^+^Ly6G^+^ immature neutrophils. CD4^+^CD25^+^ T cells had a strong correlation with unhealthy neurons and the loss of Map2^+^ neurites.

We next determined if the variance in the abnormal ranges of blood biomarkers can be predicted from the variance of neural damage due to CAR using the linear regression model for the CAR mice only while restraining the model to the average values from the sham-operated mice that have gone through the same experimental procedures as the CAR group except for the actual cardiac arrest and resuscitation manipulations. Supporting Information, Figure S4A–F depicts the linear regression, along with the goodness of fit, Sy.x, for the same biomarkers as in Figures 5 and 6. The results suggest the possibility of using circulating blood to assist in clinical evaluation of neuropathological changes after CAR.

### Fingerprint of Circulating Immunocytes after Cardiac Arrest and Resuscitation

To devise a practical strategy to use biomarkers in the circulating blood to inform CAR outcomes, the correlation analyses in Figures 5 and 6 can be used to guide the development of blood test criteria based on the experimental values separating the severely injured animals in the CAR group from the “normal values” in the naïve and sham groups. We first derived cutoff values based on ±2 SD in the direction corresponding to worse outcomes in neuron counting, Map2^+^ density, Iba1^+^ immunoreactivity, and GFAP^+^ immunoreactivity in Figures 5 and 6 using the data from the naïve and sham groups (i.e, horizontal axis values of the green and blue dots in Figures 5 and 6). We then simultaneously assigned a neuronal damage score and a neuroinflammation score to each animal based on these cutoff values. Table 1 shows a possible scoring strategy, where the neuronal damage score combines the neuronal loss (percent unhealthy neurons) and dendritic (neurite) loss (Map2^+^ density) using the “AND” and “XOR” logic operators, and the neuroinflammation score combines Iba1^+^ and GFAP^+^ immunoreactivity. Thus, an animal receives a score of 0 for neuronal damage if both neuron loss and neurite loss measures are “normal,” a score of 1 if either one of the two measures is “abnormal,” and a score of 2 if both measures are abnormal. Similarly, neuroinflammation scores of 0, 1, and 2 are based on the logic operation of the two measures for microglia and astrocyte activations. The three scores (0, 1, 2) can be categorized as normal, “mild injury,” and “severe injury”. The flow cytometry data of all animals are then grouped by the assigned neuronal damage scores and neuroinflammation scores. Table 2 summarizes subpopulations of different circulating blood cell markers that show significant differences among different scoring groups. Collectively, these biomarkers differentiating neuronal damage and neuroinflammatory scores can be used as predictive blood fingerprints for the severity of neuronal injuries and neuroinflammation after CAR. Note that a significant upregulation of the cytotoxic CD3^+^CD8^+^ subpopulation of T cells and CD4^+^CD25^+^*T*_reg_ cells, along with a concomitant downregulation of the costimulatory molecules CD80/CD86, which control T cell activation and homeostasis,^26^ are strongly associated with the severity of both neuronal damage and neural inflammation scores. Using the gating strategies as described in the Methods and Materials section, the cutoffs of CD3^+^CD8^+^ > 17%, CD4^+^CD25^+^ > 5%, and CD80^+^ < 13% and CD86^+^ < 3% would predict poor neurological outcomes after CAR.

**Table 1:** Neuronal Damage and Neuroinflammation Scoring^a^

neuronal damage			
scores	neuron loss (% injured neurons)		neurite loss (Map2^+^ pixels per neuron)
0	<10.9%	AND	>10,000
1	<10.9%	XOR	>10,000
2	≥10.9%	AND	≤10,000

neuroinflammation			
scores	Iba1 (% area)		GFAP (% area)
0	<6.5%	AND	<1.9%
1	<6.5%	XOR	<1.9%
2	≥6.5%	AND	≥1.9%

a Cutoff values were determined by two standard deviations (SDs) from the mean of the naïve and sham groups in the worse outcome direction. AND and XOR are logic operators for two measures.

**Table 2:** Circulating Immunocyte Markers Predictive of Neuronal Injury Scores^a^

cell types	injury scores	by neuronal injury categories (mean ± SEM)	by neuronal inflammation categories (mean ± SEM)	predictive cutoffs for worse outcomes
	0	15.1 ± 0.9	15.0 ± 1.0	
CD3^+^CD8^+^	1	18.9 ± 1.0	17.3 ± 1.1	>18%
	2	19.1 ± n/a	18.7 ± 1.4	
	0	3.6 ± 0.2	3.7 ± 0.2	
CD4^+^CD25^+^	1	5.2 ± 0.4	4.4 ± 0.8	>4%
	2	5.4 ± n/a	5.1 ± 0.5	
	0		16.1 ± 2.0	
CD45^+^CD11b^+^	1	NS	18.0 ± 3.2	>18%
	2		25.2 ± 2.0	
	0		13.0 ± 1.6	
CD45^+^CD11b^+^CD11c^–^	1	NS	14.9 ± 2.6	>15%
	2		21.8 ± 1.9	
	0	NS	1.1 ± 0.1	
CD11b^+^Ly6C^+^Ly6G^+^	1		1.0 ± 0.2	>1%
	2		2.3 ± 0.2	
	0	14.6 ± 1.7	14.5 ± 2.0	
CD80^+^	1	12.5 ± 2.5	13.1 ± 2.0	<13%
	2	9.8 ± n/a	11.8 ± 1.3	
	0		3.8 ± 0.9	
CD86^+^	1	NS	3.8 ± 1.5	<3%
	2		2.6 ± 0.5	
	0		40.5 ± 3.5	
F4/80	1	NS	38.8 ± 3.7	<35%
	2		25.9 ± 2.4	

a “0” indicates normal levels of injury or inflammation, e.g., in naïve and sham animals. “1” indicates moderate injury or inflammation. “2” indicates severe levels of injury or inflammation. n/a indicates a single animal in this rank. NS = not significant.

## Discussion

Blood serum biomarkers of brain injury after CAR have been reported and are quantifiable^27^ based on detection of low-level brain injury proteins that are spilled over into the bloodstream from the injured brain cells, such as neuron-specific enolase (NSE), neurofilament light chain (NFL), glia-specific calcium-binding protein B in the S-100 protein family (S100B), and GFAP. These molecules have been shown to have positive predictive values for poor outcomes after out-of-hospital cardiac arrest in the target temperature management (TTM2) trial.^27^ In this study, we focused on correlating potential blood immune cell markers to the neurologic outcomes after CAR. It has been well established that after cerebral ischemic insults, strong immune responses occur in both the CNS and the periphery.^28,29^ Using the same clinically relevant mouse cardiac arrest model as in this study, we previously characterized the time course of brain injuries, infiltration of peripheral immune cells into the brain parenchyma, and blood–brain barrier integrity 1–10 days after CAR using a combination of flow cytometry, magnetic resonance imaging, and histology approaches. In that study,^8^ we showed that the blood–brain barrier was compromised after reperfusion, allowing infiltration of peripheral myeloid-derived monocytes and DCs into the brain parenchyma. In addition to a nearly 4-fold increase in the cytotoxic CD8^+^ cells found in the brain parenchyma, which were likely infiltrating killer T cells from the blood,^30^ a profound increase in the CD45^+^CD11b^+^ monocyte and granulocyte populations were detected in the cells collected from the brain tissues 3 days after CAR.^8^ Importantly, the CD45^+hi^CD11b^+hi^ subpopulation in the brain increased more than 6-fold. This latter subpopulation was attributed to being of blood origin. In addition, we also found a 5- to 6-fold increase in the CD11b^+^CD11c^+^ DCs and CD11b^+^Ly6G^–^ monocytes in the brain. Parallel to these changes in the brain parenchyma, we found a corresponding increase in CD11b^+^CD45^+^, CD11b^+^CD11c^+^, and CD11b^+^Ly6G^–^ innate immune cells in the bone marrow and the blood 3 days after CAR. The characteristics of brain injuries and immune cell changes 3 days after CAR showed similar patterns to the data presented above for 5 days after CAR. However, we did not systematically assess the circulating T cells in the previous study, nor did we analyze the biomarker role of different subsets of immune cells in the manifestation of neuronal injuries and neuroinflammation in the previous study. Regulation of innate immune cells after CAR is likely associated with the neurological outcome, as it has been documented that immune dysfunction after CAR is linked to an increase in suppressive immune cells in out-of-hospital cardiac arrest patients.^31^ How the immune system is dysregulated after CAR and how post-CAR immunosuppression contributes to brain injuries are largely unknown.^32^ The present study shows that the different experimental manipulations in the three experimental groups lead to significantly different changes in the blood CD4^+^CD25^+^ T cells, commonly identified as immunosuppressive *T*_reg_ cells, and CD11b^+^Ly6C^+^Ly6G^+^ PMN-MDSCs, the heterogeneous granulocytic myeloid-derived cells related to immature neutrophils. The major findings are that changes in the CD4^+^CD25^+^, CD11b^+^Ly6C^+^Ly6G^+^, and CD80^+^/CD86^+^ cell populations in the circulating blood correlate strongly with neuronal injuries and neuroinflammation in the brain. No previous in vivo studies have examined these peripheral blood immune cells as biomarkers for the neuropathological processes following CAR.

The clinically relevant CAR mouse model^8^ produces characteristic ischemic brain injuries to the selectively vulnerable neurons and loss of Map2^+^ neurites in the CA1 region of the hippocampus. In the current studies using adult mice with 6 min of cardiac arrest followed by effective resuscitation, the unhealthy pyramidal neurons in the hippocampal CA1 region show large variabilities, ranging from mild to severe neuronal loss mimicking the broad neurological scores seen in human cardiac arrest patients. Moreover, the degrees of severity of neuronal damage, measured by H&E staining of unhealthy neurons and a decrease in Map2^+^ density indicating dendritic (neurite) loss, are associated with the activation of GFAP^+^ astrocytes and Iba1^+^ microglia in the hippocampal CA1 region. The role of activated microglia and astrocytes in various models of neuronal injuries has long been recognized.^28,29,33,34^ In global ischemia, the morphological changes of microglia from ramified to amoeboid shape indicate neuroinflammatory responses to the ischemic insults.^22^ It is well accepted that microglia and astrocyte activations contribute to both CNS damage and repair at different phases of injuries,^13,29,35,36^ but how neuroinflammation alters the neuropathological processes after CAR remains unclear and is likely to revolve around the CNS’s own innate immune properties. The brain tissues are sensitive to the lack of oxygenation induced by CAR. The transient anoxic condition during circulatory arrest and the protracted hypoxic condition during the critical phase of reperfusion can upregulate and trigger the release of inflammatory cytokines from the resident immune cells in the CNS. Moreover, global ischemia-mediated neuronal injury and neuroinflammation are expected to create a crosstalk between the central and peripheral immune responses for further immunocyte polarization. The results from this study suggest that the neuronal injuries and the levels of Iba1^+^ microglia and GFAP^+^ astrocytosis are tightly correlated with the increases of CD4^+^CD25^+^*T*_reg_ cells and CD11b^+^Ly6C^+^Ly6G^+^ PMN-MDSCs in the circulating blood.

In contrast to the overall maintenance of the CD3^+^ T cell population and a slight decrease in the CD4^+^ T cells (Figure 3A,B), the increase in the CD4^+^CD25^+^*T*_reg_ population correlates strongly with the neuronal and dendritic losses in the hippocampal CA1 region 5 days after CAR. The CD4^+^CD25^+^ cells are known to suppress the proliferation of other CD4^+^ and CD8^+^ T cells and are involved in immunological self-tolerance and immune suppression.^37,38^ The specific function of *T*_reg_ after cardiac arrest is unclear, but since CD4^+^CD25^+^*T*_reg_ cells play a central role in systemic immune tolerance and immune suppression, the expansion of CD4^+^CD25^+^ cells in the blood may indicate CAR-induced systemic immune suppression in response to neuronal damage and neuroinflammation. The novel finding of a positive correlation between the increase in CD4^+^CD25^+^*T*_reg_ cells and the degree of neuronal injuries and inflammation may reflect the extent to which the protective effects from the circulating immune cells can ultimately exert to mitigate the CNS damage in the critical phase of reperfusion after CAR. Hence, the results from this study suggest the possibility that quantitation of CD4^+^CD25^+^*T*_reg_ cells in the blood can be exploited as a potential clinical biomarker for outcome prognoses in cardiac arrest patients.

Only 5–9% of peripheral blood cells in mice are granulocytes, of which the large majority are neutrophils, accounting for 30–40% of the mouse white blood cells.^39^ The plasticity of these myeloid-derived populations correlates with survival and controls the body’s immune defense.^40,41^ In this study, the myeloid-derived CD11b^+^CD11c^+^ and CD11b^–^CD11c^+^ DCs and CD11b^+^Ly6C^+^Ly6G^+^ PMN-MDSCs were upregulated in the blood after CAR. PMN-MDSCs are pathologically activated neutrophils and monocytes, which play a role in the immune response to injuries. Since the CD11b^+^Ly6C^+^Ly6G^+^ cells are known to secrete IL-22 and TNFα for innate immune responses and to produce Type I interferons for mediating tissue repair,^42^ the increase in these cell populations suggests that the polarization of circulating immune cells may contribute to the innate immunity in responses to CAR-induced systemic changes. The increase of CD11b^+^Ly6C^+^Ly6G^+^ PMN-MDSCs in the blood after CAR can influence the T cell proliferation to further elevate CD4^+^CD25^+^*T*_reg_ cells. Thus, the polarization of neutrophils with the CD11b^+^Ly6C^+^Ly6G^+^ phenotype can act as myeloid-derived immunosuppressors to reinforce the action of CD4^+^CD25^+^*T*_reg_ cells. It should be noted, however, that our previous study did not identify infiltration of CD11b^+^Ly6C^+^Ly6G^+^ cells in the brain parenchyma 3 days after CAR,^8^ suggesting that the functional role of these cells in neuroimmune modulations after CAR works in a noncontact-dependent manner, possibly by limiting the damage caused by an excessive immune response while promoting tissue repair and regeneration at the same time. The exact role of the circulating CD11b^+^Ly6C^+^Ly6G^+^ PMN-MDSCs in global ischemia is still not fully understood and requires further investigation.

There have been several recent attempts to identify blood biomarkers to predict outcomes after CAR.^10,27,43,44^ In an analysis of 120 cardiac arrest patients who survived at least 48 h after the return of spontaneous circulation, angiopoietin-2 (Ang2) was the only biomarker among the eight biomarkers analyzed that had hazard ratio and discriminatory performance associated with all-cause mortality.^44^ Ang2 has long been recognized to promote *T*_reg_ cell expansion,^45^ and recent investigations have shown that after ischemic events, *T*_reg_ cells mediate angiogenesis through Ang2-dependent pathways in a tissue-specific manner by either pro- or antiangiogenesis mechanisms.^46,47^ This notion is consistent with the results from the current study showing that *T*_reg_ populations are significantly upregulated after the CAR events. It should be noted that in clinical settings, biomarker analyses necessarily consist of pooled patient data without carefully differentiating the time points after cardiac arrest. However, the consistency of the brain injury measures 3–10 days after cardiac arrest between our previous^8^ and the current studies, and the matching immune characteristics between the peripheral immune cell invasion into the brain parenchyma 3 days after CAR^8^ and the blood immune biomarkers identified here 5 days after CAR warrant future clinical trials, in which the blood from cardiac arrest patients should be analyzed for similar blood biomarker patterns as suggested in Table 2 for additional clinical measures in supplement to the standard CPC scales for better prognostication of cardiac arrest outcomes in a clinical setting.

## Conclusions

We found the upregulation of circulating immunosuppressive cells after cardiac arrest and resuscitation. In particular, CD4^+^CD25^+^*T*_reg_ cells, CD11b^+^CD11c^+^ and CD11b^–^CD11c^+^ DCs, and CD11b^+^Ly6C^+^Ly6G^+^ PMN-MDSCs are strongly correlated with neuronal injuries and neuroinflammation. The pattern of collective changes in these circulating immune cells can be exploited to devise a future strategy for the prognostication of outcomes in cardiac arrest patients. Clinical investigations to analyze blood samples from cardiac arrest patients for these biomarkers are recommended.

## Methods and Materials

### Mouse CAR Model

All animal protocols were approved by the Institutional Animal Care and Use Committees of the University of Pittsburgh and Texas Tech University. Twenty-two adult male Balb/c mice (JAX Cat. No. 000651), ∼30 g in body weight, were randomized into naïve (*n* = 6), sham (*n* = 7), and CAR (*n* = 9) groups. A clinically relevant CAR model, initially developed in rats,^24,48^ was adopted and modified for mice.^8^ After a brief anesthesia induction with 4% isoflurane, mice in the sham and CAR groups were intubated and maintained with 2% isoflurane anesthesia in 50% O_2_ and 50% air at a mechanical ventilation rate of 130 breaths/min and a tidal volume of 0.35 mL. Temperature was monitored via a tympanic temperature probe and maintained with both a regulated heating pad and an incandescent lamp, the height of which can be adjusted to maintain the mouse tympanic temperature at 36–37 °C. The left femoral artery was cannulated with Renapulse 033 tubing (Braintree Scientific) connected via a 22G Y-branching connector (Instech SCY22) to a pressure transducer for continuous monitoring of arterial blood pressure (ABP) and oxygenated blood withdrawal and infusion. The left femoral vein was cannulated with Renathane 025 tubing (Braintree Scientific) with a Y-branching connector (Instech SCY25) for the administration of esmolol, a short-acting β-adrenergic blocker, for the rapid and reversible induction of cardiac arrest. After the establishment of the lines, ∼300 μL of arterial blood was withdrawn, of which ∼100 μL was used for blood gas analysis. The remaining oxygenated blood (200 μL) was mixed with 20 μL of a stock solution of a resuscitation mixture, composed of epinephrine (0.4 mg/mL), sodium bicarbonate (0.5 mEq/mL), and heparin (50 U/mL) in physiological saline. Five minutes before the induction of cardiac arrest, complete muscle relaxation was achieved by subcutaneous administration of 1 mg/kg vecuronium bromide to prevent spontaneous breathing during cardiac arrest. CAR was initiated by an intravenous infusion of 70–80 μL of 30 mg/mL esmolol, causing a rapid onset of electromechanical disassociation within 30 ± 10 s. Mechanical ventilation was stopped to ensure a steady decrease of ABP below 9 mmHg and the pulse pressure below 1 mmHg, which marked the beginning of cardiac arrest. The duration of cardiac arrest was precisely controlled to 6 min before resuscitation was initiated by restarting the ventilator with 100% O_2_ without isoflurane and a slow retrograde infusion of the oxygenated blood containing the resuscitation mixture through the arterial cannulas over the course of 1–2 min until the return of spontaneous circulation was observed. Mice were allowed to recover with mechanical ventilation for 1.5–2 h, during which isoflurane anesthesia was gradually reinstated, the cannula was removed, the surgical wound was closed, and oxygen content in ventilation was gradually reduced from 100 to 21% (air). Before returning them to the colony, the mice were given 5 mg/kg ropivacaine at the surgical site. The same anesthesia and surgical procedures were performed in the sham group, except for the CAR steps. Sham and CAR mice were sacrificed 5 days after the procedure. Age-matched naïve mice had no surgical manipulation prior to sacrifice.

### Tissue Preparation

Five days after the CAR or sham operation, mice (including age-matched naïve controls) were sacrificed via isoflurane overdose. After cessation of breathing and absence of reaction to strong toe pinches but before the heartbeat stopped, the chest was opened, and approximately 1 mL of blood was drawn from the right ventricle. This blood was mixed with 50 μL of heparin, diluted 1:1 with phosphate-buffered saline (PBS), and carefully layered over 5 mL of Lympholyte Mammal Cell Separation Media (Cedarlane Laboratories). The blood and density separation medium were centrifuged at 800*g* and 4 °C for 30 min without brake during deceleration. After centrifugation, serum samples were removed from the top layer, flash-frozen in liquid nitrogen, and stored at −80 °C until further analyses. The interface between the serum and the separation media was carefully collected, passed through a 40 μm mesh, and rinsed with PBS. These cells were kept on ice, while the rest of the tissue was processed.

While the blood was centrifuged, the animal brain was carefully extracted, transaxially cut into a single section containing the hippocampus, and placed in 4% paraformaldehyde (PFA) at 4 °C for 24 h. Thereafter, the brain was transferred to 70% ethanol and kept at 4 °C until the time for paraffin embedding.

The spleen cells were harvested to provide single-dye controls for the viability stain. The spleen was removed, kept on ice in Roswell Park Memorial Institute (RPMI) 1640 media with Glutamax, HEPES (Fisher Cat 72-400-047), and 5% fetal bovine serum, mashed through a 40 μm sterile mesh with a sterile plunger, rinsed with additional RPMI + HEPES, and kept on ice until further processing.

After tissue collection, the blood and spleen cells were centrifuged at 400*g* and 4 °C for 5 min, and the supernatants were discarded. Each cell type was separately resuspended in ammonium chloride potassium (ACK) lysing buffer to lyse red blood cells. After incubating for 2 min in a 37 °C water bath, the reactions were quenched with 2× volumes of cold PBS. All cells were centrifuged again at 400*g* and 4 °C for 5 min before being prepared for flow cytometry (see below).

### Histological Evaluation

Standard protocols were used for hematoxylin and eosin (H&E) staining and immunohistochemistry staining.^22^ Briefly, brain tissues collected 5 days after CAR were embedded in paraffin and cut into 6 μm sections. H&E staining was performed by the Research Histology Services at University of Pittsburgh. For fluorescence staining, microscope-slide-mounted tissue sections were baked at 56 °C for 60 min, deparaffinized/rehydrated, boiled in antigen retrieval buffer (10 mM sodium citrate, 0.05% Tween 20, pH 6) for 30 min, and allowed to cool for at least 30 min to room temperature. After incubation in blocking buffer (5% goat serum, 1% bovine serum albumin, and 0.2% Triton X-100 in PBS, pH 7.4) at room temperature for 30 min, primary antibodies, diluted in blocking buffer according to Supporting Information, Table S1, were applied. Glial fibrillary acidic protein (GFAP) and ionized calcium-binding adaptor molecule 1 (Iba1) were costained, as were microtubule-associated protein 2 (Map2) and neuronal nuclear marker (Neun). After incubation with the primary antibodies overnight at 4 °C, slides were washed five times for 5 min each with PBS-T (PBS with 0.1% Tween 20, pH 7.4). Secondary antibodies (Alexa 594 Goat anti-Rabbit and Alexa 488 Goat anti-Mouse IgG1, both from Invitrogen) were diluted 1:500 in blocking buffer and incubated with the tissue in the dark at room temperature for 2 h. After five washes with PBS-T, the slides were coverslipped with VectaShield HardSet mounting media (Vector Laboratories). The H&E and fluorescent sections were imaged using an Olympus IX81 microscope equipped with a Q-Imaging Retiga-2000R color camera (Teledyne Photometrics, Tucson, AZ) and a Hamamatsu ORCA-ER monochromatic camera (Hamamatsu Co., Bridgewater, NJ).

All acquired images were assigned generic codes to blind the experimental groups, and the hippocampi were then divided into four equally sized circular ROIs, medially to laterally along Cornu Ammonis region 1 (CA1) of the hippocampi. Each ROI encompasses the pyramidal neuron band containing ∼100–200 pyramidal neurons. Neurons in the ROIs were evaluated independently by three trained investigators and marked as healthy or unhealthy. Unhealthy neuronal markers include strong basophilic staining, vacuolization, pyknosis, and karyorrhexis.^24^ Experimenters also looked for cell morphology and differing colorization of cells as further evidence of declining cell health or cell death. A small number of cells showing clear embedding artifacts were excluded from the healthy–unhealthy categorization. Unhealthy and healthy cells were then quantified in each of the four ROIs and pulled together to obtain a total percentage of unhealthy neurons. For Iba1 and GFAP staining, ROI encompassed the CA1 region extending from the dentate gyrus to the corpus callosum with 36 ± 22 million total pixels. The image intensities were processed by thresholding segmentation using SlideBook version 6.21 (Intelligent Imaging Innovations, Inc., Denver, CO), and the total number of positively stained pixels within the ROI was divided by the total number of pixels in the region to quantify the percent positive staining areas. For Map2 and Neun quantification, the ROI encompassed comparable CA1 bands in all tissue sections, extending from the hippocampal fissure to the nuclei of the pyramidal neurons. The number of Neun positive cells within the bands was estimated by dividing the total Neun positive area (in pixels) by the average size of a neuronal nucleus (in pixels) and verified by manual counting in randomly selected sections. To quantify Map2, the same segmentation method as that for GFAP and Iba1 was used to quantify the area of positive Map2 staining. For each mouse, the Map2^+^ area in pixels was divided by the number of neuronal nuclei from the NeuN costaining to normalize the dendritic processes per CA1 neuron.

### Antibody Panels to Identify Circulating Blood Immunocytes

To identify blood immune cells and their subpopulations in the animals after CAR, two panels of immune cell markers were selected, as shown in Table S2, for labeling blood lymphocytes and for identifying blood myeloid cells, respectively. Optimization of antibody concentrations was performed by serial dilutions as per the manufacturer’s recommended procedures, and the optimized antibody dilutions are listed in Table S2. White blood cells from each mouse were divided into two equal portions for flow cytometry with the two antibody panels.

### Flow Cytometry Assay

Cells were first stained in the dark with 100 μL of viability dye diluted 1:500 in PBS at room temperature for 30 min. Staining was stopped by adding 2 mL of fluorescence-activated cell sorting (FACS) buffer (1% BSA, 5 mM EDTA, and 0.05% sodium azide in PBS). After centrifugation, the cells were resuspended in 100 μL of antibody mixtures for the immune cell surface markers from each panel. Antibodies were diluted at the dilution ratios listed in Table S2 using a 1:1 mixture of FACS buffer and Brilliant Buffer (BD Biosciences). Cells were stained in the dark at room temperature for 1 h. Staining was stopped by dilution in 2 mL of FACS buffer. After centrifugation, all pellets were fixed with 2% paraformaldehyde (PFA) in the dark for 30 min. At this point, Panel 1 samples were centrifuged and washed with 0.5% saponin in FACS buffer and centrifuged again before resuspending with FoxP3 and IFNγ antibodies diluted in 0.5% saponin in FACS buffer. After incubation at room temperature and in the dark for 1 h, cells were collected and resuspended in FACS buffer. Samples were kept at 4 °C in the dark until the flow cytometry experiments.

Single-dye controls were prepared using BD CompBeads (BD Biosciences) or VersaComp antibody capture beads (Beckman Coulter Life Sciences) stained according to the manufacturer’s directions. For viability dye, spleen cells were used as the positive control instead of beads. Single-dye controls were acquired for each set of flow cytometry data. All samples were run on the same LSR II flow cytometry machine at the Unified Flow Core at the University of Pittsburgh and processed using FlowJo 10.7.1 (Becton Dickinson & Co.).

### Statistical Analysis

Comparison among the three experimental groups was carried out using the one-way ANOVA design with Fisher’s LSD post hoc test. Statistical significance was set at *p* < 0.05, with bar graphs showing mean ± SEM. Pearson correlation coefficients were calculated with the statistical significance set at a two-sided *p*-value of <0.05. Simple linear regression lines were plotted for significant correlations only. Results are reported in the text as mean ± SEM.

## Data Availability

Raw data and all related materials are available upon request from the corresponding authors.

## Supporting Information Available

List of antibodies for immunohistochemistry and flow cytometry; gating strategies for isolating blood lymphocytes and monocytes; NeuN^+^ and MAP2^+^ threshold segmentations for quantitation in Figure 2D; and relationship between neuronal injuries and blood surface markers in CAR mice (PDF)

## References

[ref1] Berdowski J.; Berg R. A.; Tijssen J. G.; Koster R. W. Global incidences of out-of-hospital cardiac arrest and survival rates: Systematic review of 67 prospective studies. Resuscitation 2010, 81 (11), 1479–1487. 10.1016/j.resuscitation.2010.08.006. 20828914

[ref2] Brady W. J.; Gurka K. K.; Mehring B.; Peberdy M. A.; O’Connor R. E.; In-hospital cardiac arrest: impact of monitoring and witnessed event on patient survival and neurologic status at hospital discharge. Resuscitation 2011, 82 (7), 845–852. 10.1016/j.resuscitation.2011.02.028. 21454008

[ref3] Majewski D.; Ball S.; Bailey P.; McKenzie N.; Bray J.; Morgan A.; Finn J. Survival to hospital discharge is equivalent to 30-day survival as a primary survival outcome for out-of-hospital cardiac arrest studies. Resuscitation 2021, 166, 43–48. 10.1016/j.resuscitation.2021.07.023. 34314779

[ref4] Olsen J. A.; Brunborg C.; Steinberg M.; Persse D.; Sterz F.; Lozano M. Jr.; Westfall M.; van Grunsven P. M.; Lerner E. B.; Wik L. Survival to hospital discharge with biphasic fixed 360 joules versus 200 escalating to 360 joules defibrillation strategies in out-of-hospital cardiac arrest of presumed cardiac etiology. Resuscitation 2019, 136, 112–118. 10.1016/j.resuscitation.2019.01.020. 30708074

[ref5] Yan S.; Gan Y.; Jiang N.; Wang R.; Chen Y.; Luo Z.; Zong Q.; Chen S.; Lv C. The global survival rate among adult out-of-hospital cardiac arrest patients who received cardiopulmonary resuscitation: a systematic review and meta-analysis. Crit. Care 2020, 24 (1), 61 10.1186/s13054-020-2773-2. 32087741 PMC7036236

[ref6] Sandroni C.; Geocadin R. G. Neurological prognostication after cardiac arrest. Curr. Opin. Crit. Care 2015, 21 (3), 209–214. 10.1097/MCC.0000000000000202. 25922894 PMC4955580

[ref7] Sandroni C.; D’Arrigo S.; Nolan J. P. Prognostication after cardiac arrest. Crit. Care 2018, 22 (1), 150 10.1186/s13054-018-2060-7. 29871657 PMC5989415

[ref8] Zhang C.; Brandon N. R.; Koper K.; Tang P.; Xu Y.; Dou H. Invasion of Peripheral Immune Cells into Brain Parenchyma after Cardiac Arrest and Resuscitation. Aging Dis. 2018, 9 (3), 412–425. 10.14336/AD.2017.0926. 29896429 PMC5988596

[ref9] Beurskens C. J.; Horn J.; de Boer A. M.; Schultz M. J.; van Leeuwen E. M.; Vroom M. B.; Juffermans N. P. Cardiac arrest patients have an impaired immune response, which is not influenced by induced hypothermia. Crit. Care 2014, 18 (4), R162 10.1186/cc14002. 25078879 PMC4261599

[ref10] Qi Z.; Zhang Q.; Liu B.; Shao F.; Li C. Presepsin As a Biomarker for Evaluating Prognosis and Early Innate Immune Response of Out-of-Hospital Cardiac Arrest Patients After Return of Spontaneous Circulation. Crit. Care Med. 2019, 47 (7), e538–e546. 10.1097/CCM.0000000000003764. 30985453

[ref11] Tsivilika M.; Doumaki E.; Stavrou G.; Sioga A.; Grosomanidis V.; Meditskou S.; Maranginos A.; Tsivilika D.; Stafylarakis D.; Kotzampassi K.; Papamitsou T. The adaptive immune response in cardiac arrest resuscitation induced ischemia reperfusion renal injury. J. Biol. Res. 2020, 27, 15 10.1186/s40709-020-00125-2. PMC7526263 33014901

[ref12] Ferguson M. A.; Sutton R. M.; Karlsson M.; Sjovall F.; Becker L. B.; Berg R. A.; Margulies S. S.; Kilbaugh T. J. Increased platelet mitochondrial respiration after cardiac arrest and resuscitation as a potential peripheral biosignature of cerebral bioenergetic dysfunction. J. Bioenerg. Biomembr. 2016, 48 (3), 269–279. 10.1007/s10863-016-9657-9. 27020568

[ref13] Dokalis N.; Prinz M. Resolution of neuroinflammation: mechanisms and potential therapeutic option. Semin. Immunopathol. 2019, 41 (6), 699–709. 10.1007/s00281-019-00764-1. 31705317

[ref14] Tahsili-Fahadan P.; Farrokh S.; Geocadin R. G. Hypothermia and brain inflammation after cardiac arrest. Brain Circ. 2018, 4 (1), 1–13. 10.4103/bc.BC_4_18. 30276330 PMC6057700

[ref15] Anrather J.; Iadecola C. Inflammation and Stroke: An Overview. Neurotherapeutics 2016, 13 (4), 661–670. 10.1007/s13311-016-0483-x. 27730544 PMC5081118

[ref16] Famakin B. M. The Immune Response to Acute Focal Cerebral Ischemia and Associated Post-stroke Immunodepression: A Focused Review. Aging Dis. 2014, 5 (5), 307–326. 10.14336/ad.2014.0500307. 25276490 PMC4173797

[ref17] Xu Y.; Liachenko S. M.; Tang P.; Chan P. H. Faster recovery of cerebral perfusion in SOD1-overexpressed rats after cardiac arrest and resuscitation. Stroke 2009, 40 (7), 2512–2518. 10.1161/STROKEAHA.109.548453. 19461023 PMC2919566

[ref18] Mölström S.; Nielsen T. H.; Nordstrom C. H.; Forsse A.; M?ller S.; Veno S.; Mamaev D.; Tencer T.; Schmidt H.; Toft P. Bedside microdialysis for detection of early brain injury after out-of-hospital cardiac arrest. Sci. Rep. 2021, 11 (1), 15871 10.1038/s41598-021-95405-9. 34354178 PMC8342553

[ref19] Shinozaki K.; Becker L. B.; Saeki K.; Kim J.; Yin T.; Da T.; Lampe J. W. Dissociated Oxygen Consumption and Carbon Dioxide Production in the Post-Cardiac Arrest Rat: A Novel Metabolic Phenotype. J. Am. Heart Assoc. 2018, 7 (13), e007721 10.1161/JAHA.117.007721. 29959138 PMC6064898

[ref20] Wei Z.; Wang Q.; Modi H. R.; Cho S. M.; Geocadin R.; Thakor N. V.; Lu H. Acute-stage MRI cerebral oxygen consumption biomarkers predict 24-h neurological outcome in a rat cardiac arrest model. NMR Biomed. 2020, 33 (11), e4377 10.1002/nbm.4377. 32662593 PMC7541582

[ref21] Hosseini M.; Wilson R. H.; Crouzet C.; Amirhekmat A.; Wei K. S.; Akbari Y. Resuscitating the Globally Ischemic Brain: TTM and Beyond. Neurotherapeutics 2020, 17 (2), 539–562. 10.1007/s13311-020-00856-z. 32367476 PMC7283450

[ref22] Hirko A. C.; Dallasen R.; Jomura S.; Xu Y. Modulation of inflammatory responses after global ischemia by transplanted umbilical cord matrix stem cells. Stem Cells 2008, 26 (11), 2893–2901. 10.1634/stemcells.2008-0075. 18719227 PMC2802492

[ref23] Jomura S.; Uy M.; Mitchell K.; Dallasen R.; Bode C. J.; Xu Y. Potential treatment of cerebral global ischemia with Oct-4+ umbilical cord matrix cells. Stem Cells 2007, 25 (1), 98–106. 10.1634/stemcells.2006-0055. 16960128

[ref24] Liachenko S.; Tang P.; Hamilton R. L.; Xu Y. A reproducible model of circulatory arrest and remote resuscitation in rats for NMR investigation. Stroke 1998, 29 (6), 1229–1238. 10.1161/01.STR.29.6.1229. 9626299

[ref25] Neigh G. N.; Glasper E. R.; Bilbo S. D.; Traystman R. J.; Courtney DeVries A. Cardiac arrest/cardiopulmonary resuscitation augments cell-mediated immune function and transiently suppresses humoral immune function. J. Cereb. Blood Flow Metab. 2005, 25 (11), 1424–1432. 10.1038/sj.jcbfm.9600137. 15874972

[ref26] Trzupek D.; Dunstan M.; Cutler A. J.; Lee M.; Godfrey L.; Jarvis L.; Rainbow D. B.; Aschenbrenner D.; Jones J. L.; Uhlig H. H.; Wicker L. S.; Todd J. A.; Ferreira R. C. Discovery of CD80 and CD86 as recent activation markers on regulatory T cells by protein-RNA single-cell analysis. Genome Med. 2020, 12 (1), 55 10.1186/s13073-020-00756-z. 32580776 PMC7315544

[ref27] Moseby-Knappe M.; Mattsson-Carlgren N.; Stammet P.; Backman S.; Blennow K.; Dankiewicz J.; Friberg H.; Hassager C.; Horn J.; Kjaergaard J.; Lilja G.; Rylander C.; Ullen S.; Unden J.; Westhall E.; Wise M. P.; Zetterberg H.; Nielsen N.; Cronberg T. Serum markers of brain injury can predict good neurological outcome after out-of-hospital cardiac arrest. Intensive Care Med. 2021, 47 (9), 984–994. 10.1007/s00134-021-06481-4. 34417831 PMC8421280

[ref28] Pekny M.; Wilhelmsson U.; Pekna M. The dual role of astrocyte activation and reactive gliosis. Neurosci. Lett. 2014, 565, 30–38. 10.1016/j.neulet.2013.12.071. 24406153

[ref29] Xu S.; Lu J.; Shao A.; Zhang J. H.; Zhang J. Glial Cells: Role of the Immune Response in Ischemic Stroke. Front. Immunol. 2020, 11, 294 10.3389/fimmu.2020.00294. 32174916 PMC7055422

[ref30] Smida T.; Koller A. C.; Menegazzi J. J.; Salcido D. D. Early cytotoxic lymphocyte localization to the brain following resuscitation in a porcine model of asphyxial cardiac arrest: A pilot study. Resusc. Plus. 2021, 6, 100125 10.1016/j.resplu.2021.100125. 34223383 PMC8244478

[ref31] Qi Z.; Liu Q.; Zhang Q.; Liu B.; Li C. Overexpression of programmed cell death-1 and human leucocyte antigen-DR on circulatory regulatory T cells in out-of-hospital cardiac arrest patients in the early period after return of spontaneous circulation. Resuscitation 2018, 130, 13–20. 10.1016/j.resuscitation.2018.06.023. 29940295

[ref32] Zhao Q.; Shen Y.; Li R.; Wu J.; Lyu J.; Jiang M.; Lu L.; Zhu M.; Wang W.; Wang Z.; Liu Q.; Hoffmann U.; Karhausen J.; Sheng H.; Zhang W.; Yang W. Cardiac arrest and resuscitation activates the hypothalamic-pituitary-adrenal axis and results in severe immunosuppression. J. Cereb. Blood Flow Metab. 2021, 41 (5), 1091–1102. 10.1177/0271678X20948612. 32787543 PMC8054717

[ref33] Devanney N. A.; Stewart A. N.; Gensel J. C. Microglia and macrophage metabolism in CNS injury and disease: The role of immunometabolism in neurodegeneration and neurotrauma. Exp. Neurol. 2020, 329, 113310 10.1016/j.expneurol.2020.113310. 32289316 PMC7237336

[ref34] Dong R.; Huang R.; Wang J.; Liu H.; Xu Z. Effects of Microglial Activation and Polarization on Brain Injury After Stroke. Front. Neurol. 2021, 12, 620948 10.3389/fneur.2021.620948. 34276530 PMC8280287

[ref35] Sandvig I.; Augestad I. L.; Haberg A. K.; Sandvig A. Neuroplasticity in stroke recovery. The role of microglia in engaging and modifying synapses and networks. Eur. J. Neurosci. 2018, 47 (12), 1414–1428. 10.1111/ejn.13959. 29786167

[ref36] Yong H. Y. F.; Rawji K. S.; Ghorbani S.; Xue M.; Yong V. W. The benefits of neuroinflammation for the repair of the injured central nervous system. Cell. Mol. Immunol. 2019, 16 (6), 540–546. 10.1038/s41423-019-0223-3. 30874626 PMC6804643

[ref37] Giganti G.; Atif M.; Mohseni Y.; Mastronicola D.; Grageda N.; Povoleri G. A.; Miyara M.; Scotta C. Treg cell therapy: How cell heterogeneity can make the difference. Eur. J. Immunol. 2021, 51 (1), 39–55. 10.1002/eji.201948131. 33275279

[ref38] Saxena V.; Lakhan R.; Iyyathurai J.; Bromberg J. S. Mechanisms of exTreg induction. Eur. J. Immunol. 2021, 51 (8), 1956–1967. 10.1002/eji.202049123. 33975379 PMC8338747

[ref39] O’Connell K. E.; Mikkola A. M.; Stepanek A. M.; Vernet A.; Hall C. D.; Sun C. C.; Yildirim E.; Staropoli J. F.; Lee J. T.; Brown D. E. Practical murine hematopathology: a comparative review and implications for research. Comp. Med. 2015, 65 (2), 96–113. 25926395 PMC4408895

[ref40] Ryzhov S.; May T.; Dziodzio J.; Emery I. F.; Lucas F. L.; Leclerc A.; McCrum B.; Lord C.; Eldridge A.; Robich M. P.; Ichinose F.; Sawyer D. B.; Riker R.; Seder D. B. Number of Circulating CD 73-Expressing Lymphocytes Correlates With Survival After Cardiac Arrest. J. Am. Heart Assoc. 2019, 8 (13), e010874 10.1161/JAHA.118.010874. 31237169 PMC6662342

[ref41] Torosyan N.; Gonzalez Mancera M. S.; Tourtellotte W. G.; Kedan I. Leukemic Infiltration of Myocardium Presenting as Cardiac Arrest. JACC Case Rep. 2021, 3 (6), 922–927. 10.1016/j.jaccas.2021.01.033. 34317656 PMC8311278

[ref42] Fischer M. A.; Davies M. L.; Reider I. E.; Heipertz E. L.; Epler M. R.; Sei J. J.; Ingersoll M. A.; Rooijen N. V.; Randolph G. J.; Norbury C. C. CD11b(+), Ly6G(+) cells produce type I interferon and exhibit tissue protective properties following peripheral virus infection. PLoS Pathog. 2011, 7 (11), e1002374 10.1371/journal.ppat.1002374. 22102816 PMC3213107

[ref43] Wihersaari L.; Ashton N. J.; Reinikainen M.; Jakkula P.; Pettila V.; Hastbacka J.; Tiainen M.; Loisa P.; Friberg H.; Cronberg T.; Blennow K.; Zetterberg H.; Skrifvars M. B.; Comacare Study G.; et al. Neurofilament light as an outcome predictor after cardiac arrest: a post hoc analysis of the COMACARE trial. Intensive Care Med. 2021, 47 (1), 39–48. 10.1007/s00134-020-06218-9. 32852582 PMC7782453

[ref44] Zelniker T. A.; Kaya Z.; Gamerdinger E.; Spaich S.; Stiepak J.; Giannitsis E.; Katus H. A.; Preusch M. R. Relationship between markers of inflammation and hemodynamic stress and death in patients with out-of-hospital cardiac arrest. Sci. Rep. 2021, 11 (1), 9954 10.1038/s41598-021-88474-3. 33976254 PMC8113496

[ref45] Coffelt S. B.; Chen Y. Y.; Muthana M.; Welford A. F.; Tal A. O.; Scholz A.; Plate K. H.; Reiss Y.; Murdoch C.; De Palma M.; Lewis C. E. Angiopoietin 2 stimulates TIE2-expressing monocytes to suppress T cell activation and to promote regulatory T cell expansion. J. Immunol. 2011, 186 (7), 4183–4190. 10.4049/jimmunol.1002802. 21368233

[ref46] Lužnik Z.; Anchouche S.; Dana R.; Yin J. Regulatory T Cells in Angiogenesis. J. Immunol. 2020, 205 (10), 2557–2565. 10.4049/jimmunol.2000574. 33168598 PMC7664842

[ref47] Zhu H.; Zhang Y.; Zhong Y.; Ye Y.; Hu X.; Gu L.; Xiong X. Inflammation-Mediated Angiogenesis in Ischemic Stroke. Front. Cell. Neurosci. 2021, 15, 652647 10.3389/fncel.2021.652647. 33967696 PMC8096981

[ref48] Liachenko S.; Tang P.; Hamilton R. L.; Xu Y. Regional dependence of cerebral reperfusion after circulatory arrest in rats. J. Cereb. Blood Flow Metab. 2001, 21 (11), 1320–1329. 10.1097/00004647-200111000-00008. 11702047

