# Supplementary material for: Fingerprint of Circulating Immunocytes as Biomarkers for the Prognosis of Brain Inflammation and Neuronal Injury after Cardiac Arrest
Source: ACS Chem Neurosci. 2023 Nov 15;14(23):4115–27. doi: 10.1021/acschemneuro.3c00397 (PMC10704468; doi:10.1021/acschemneuro.3c00397)
Supplement: Supplementary file 1 — cn3c00397_si_001.pdf [file cn3c00397_si_001.pdf]

SUPPORTING INFORMATION

Fingerprint of circulating immunocytes as biomarkers for prognosis of brain inflammation and neuronal injury after cardiac arrest

Running title: Biomarkers for neuropathology after cardiac arrest

Huanyu Dou,[1*] Nicole R. Brandon,[2] Kerryann E. Koper,[2] and Yan Xu[2,3,*]

[1]Department of Molecular and Translational Medicine, Paul L. Foster School of Medicine, and Graduate School of Biomedical Sciences, Texas Tech University Health Science Center, El Paso, Texas 79905, USA

[2]Departments of Anesthesiology and Perioperative Medicine, Pharmacology and Chemical Biology, and Structural Biology, University of Pittsburgh School of Medicine, Pittsburgh, Pennsylvania 15213, USA

[3]Department of Physics and Astronomy, The Dietrich School of Arts and Sciences, University of Pittsburgh, Pittsburgh, Pennsylvania 15213, USA

Table S1. Primary antibodies used for immunohistochemistry staining

Primary Antibody	Host	Dilution	Company
GFAP	Mouse IgG1	1:400	US Biologicals
Iba1	Rabbit	1:200	Thermofisher Scientific
Map2	Rabbit	1:200	Sigma/Millipore
NeuN	Mouse IgG1	1:500	Sigma/Millipore

Table S2. Antibody panels for flow cytometry analyses of immune cell markers in the blood

Panel 1			Panel 2		
**Antibody**	**Dye**	**Dilution**	**Antibody**	**Dye**	**Dilution**
CD3e	V450	1:200	CD11b	BUV395	1:200
CD4	PerCP-Cy5.5	1:200	CD11c	APC	1:100
CD8a	BUV395	1:200	CD45	APC-Cy7	1:200
CD25	PE-Cy7	1:200	CD80	FITC	1:200
CD28	PE	1:100	CD86	V450	1:200
CD45	APC-Cy7	1:200	F4/80	PE	1:200
FoxP3	Alexa Fluor 647	1:200	Ly-6C	PerCP-Cy5.5	1:200
IFNγ	FITC	1:200	Ly-6G	PE-eFluor610	1:200
Viability (Live/Dead Fixable Yellow)	405ex/575em	1:500	Viability (Live/Dead Fixable Yellow)	405ex/575em	1:500

Figure S1

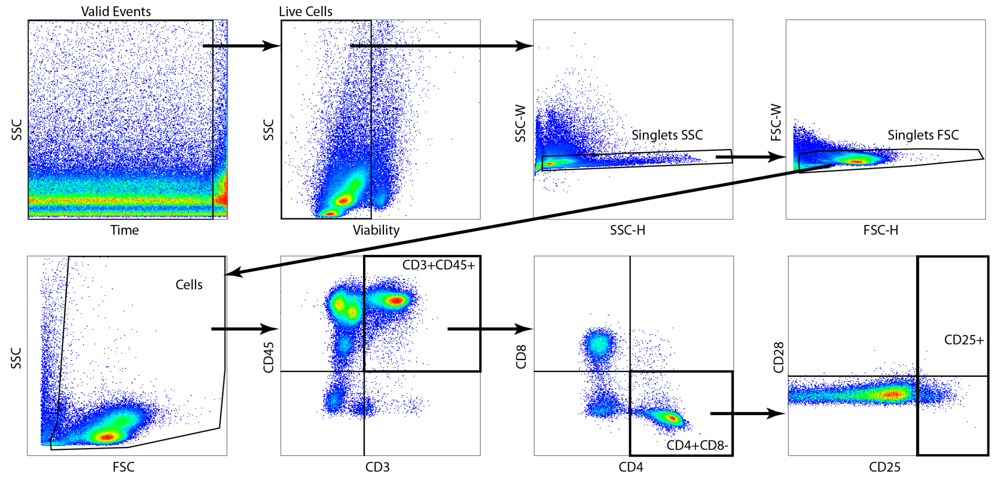

Figure S1. Gating strategy to isolate circulating blood lymphocytes. Representative gating strategy to identify T cells. The identified cell populations are plotted to display all $CD45^{+}CD3^{+}$ T cells. The entire population of the $CD45^{+}CD3^{+}$ T cells are then plotted to group $CD4^{+}$ and $CD8^{+}$ subpopulations. Sub-gating of the $CD4^{+}CD8^{-}$ T cells by CD28 and CD25 allows for quantification of $CD4^{+}CD25^{+}$ T cells.

Figure S2.

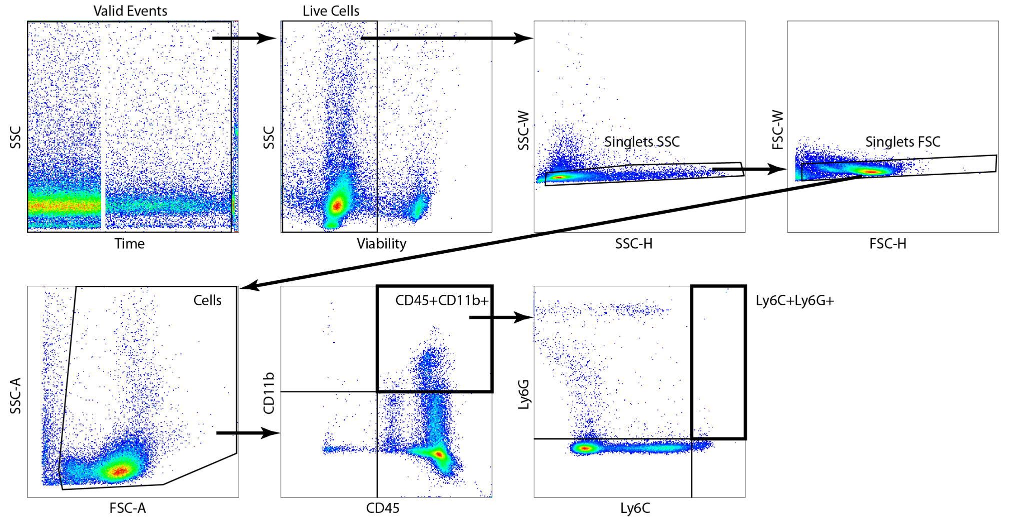

Figure S2. Gating strategy to isolate circulating blood monocytes. Representative gating strategy to identify monocytes. $CD45^{+}CD11b^{+}$ populations are plotted to display monocyte populations. All $CD45^{+}CD11b^{+}$ cells are then plotted to separate $Ly6C^{+}$, $Ly6G^{+}$, and $Ly6C^{+}Ly6G^{+}$ sub-populations.

Figure S3

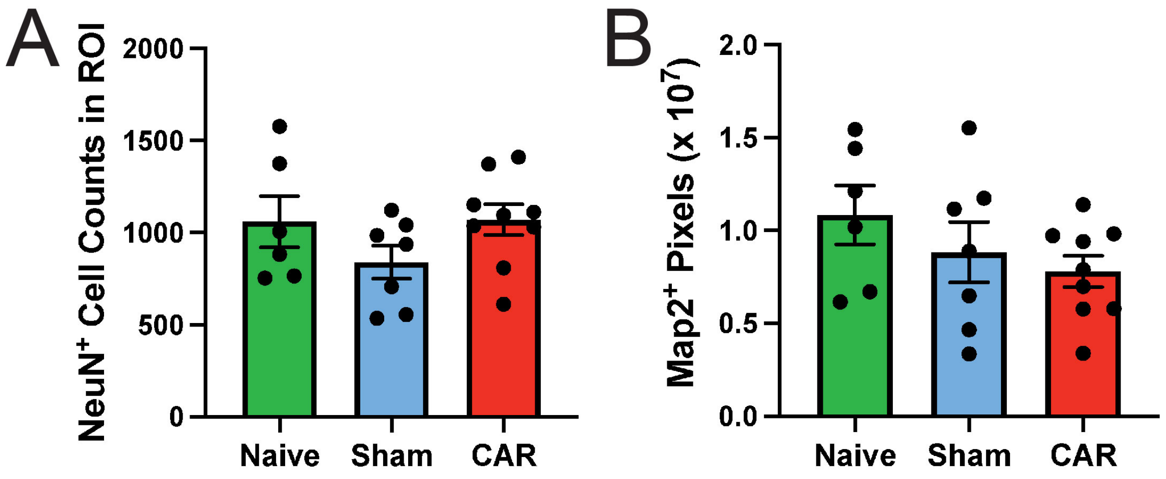

Figure S3. Quantitative analysis of dendritic loss in the hippocampal CA1 region. **(A)** Unlike H&E staining that identifies unhealthy neurons as shown in Figures 2A and 2C, the neuronal nuclear staining by NeuN reveals nuclei of both healthy and injured neurons (Figures 2B green). To quantify dendritic loss in the neuronal processes, a comparable number of neurons is needed to normalize the dendritic densities. Shown here are the $NeuN^+$ cell counts in the region of interest (ROI) from three experimental groups. **(B)** In the ROI of the hippocampal CA1 area extending from the hippocampal fissure to the nuclei of the pyramidal neurons, the $Map2^+$ pixels using the threshold segmentation method are displayed for the three experimental groups. Each black dot in (A) and (B) represents one animal. The corresponding ratios of B/A from each individual animal are used to quantify dendritic loss per neuron as presented in Figure 2D.

Figure S4

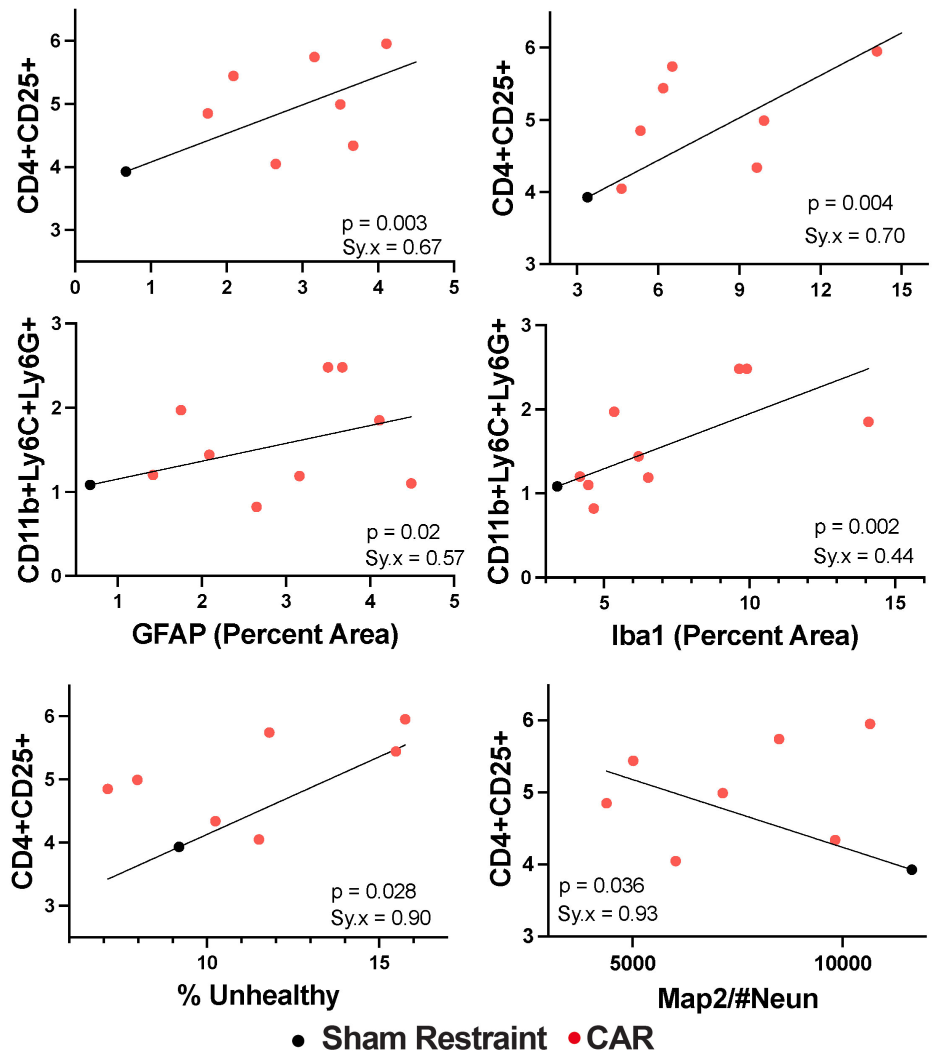

Figure S4. Linear regression model for prediction of variance in the abnormal ranges of blood biomarkers from the variance of neuronal damage measures. The plotted panels correspond to those in Figures 5 and 6 but use the CAR group only, with linear regression model restrained to the average values (black dots) from the Sham group, which has the same experimental procedures except for the actual cardiac arrest manipulations. Sy.x indicates goodness of fit.
